# Hippocampus Leads Ventral Striatum in Replay of Place-Reward Information

**DOI:** 10.1371/journal.pbio.1000173

**Published:** 2009-08-18

**Authors:** Carien S. Lansink, Pieter M. Goltstein, Jan V. Lankelma, Bruce L. McNaughton, Cyriel M. A. Pennartz

**Affiliations:** 1 Swammerdam Institute for Life Sciences–Center for Neuroscience, University of Amsterdam, Amsterdam, the Netherlands; 2 Canadian Center for Behavioral Neuroscience, The University of Lethbridge, Lethbridge, Canada; Salk Institute for Biological Studies, United States of America

## Abstract

Associating spatial locations with rewards is fundamental to survival in natural environments and requires the integrity of the hippocampus and ventral striatum. In joint multineuron recordings from these areas, hippocampal–striatal ensembles reactivated together during sleep. This process was especially strong in pairs in which the hippocampal cell processed spatial information and ventral striatal firing correlated to reward. Replay was dominated by cell pairs in which the hippocampal “place” cell fired preferentially before the striatal reward-related neuron. Our results suggest a plausible mechanism for consolidating place-reward associations and are consistent with a central tenet of consolidation theory, showing that the hippocampus leads reactivation in a projection area.

## Abstract 2

While the brain is asleep, the hippocampus plays first fiddle in the orchestra of memory; spatial information is reactivated in the hippocampus shortly in advance of emotional memory traces in the vental striatum.

## Author Summary

Thinking back to an exciting event often includes the scene in which the event took place. Associations between specific places and emotional events are consolidated in memory, but how this is achieved is currently unknown. Two brain areas involved in learning such associations are the hippocampus and the ventral striatum, which represent spatial and emotional information, respectively. A highly valuable object in an environment will prompt humans and animals to take action, such as approaching the object. Here, we demonstrate that a combination of spatial and emotional aspects of a learning experience is replayed in the hippocampus and the ventral striatum during sleep, which is likely to contribute to the consolidation and strengthening of memory traces. This reactivation is coordinated such that the spatial information in the hippocampus is activated shortly before the emotional information in the ventral striatum. This finding is consistent with a central prediction from Memory Consolidation Theory, namely that the hippocampus initiates and orchestrates replay in connected brain areas. In addition, sleep replay occurs at a time scale about ten times faster than during the actual experience, which makes it a mechanism suitable for strengthening synaptic connections associating place with reward. Our results shed new light on the distributed way the brain processes, links, and retrieves different aspects of memories.

## Introduction

Successful foraging requires that animals maintain a representation of a multitude of reward properties including the location at which a reward can be found. Forming a place–reward association is thought to depend critically on the communication between the hippocampal formation and the ventral striatum (VS). Cells in the hippocampus proper (HC) [1],[2] and adjacent subiculum [3] show location-specific firing (i.e., “place fields”), and these structures are crucial for spatial and contextual learning [2],[4]–[6]. Neurons in the VS fire in relation to rewards, as they are expected or actually delivered, as well as to cues predictive of reward [7]–[9]. Receiving information from a range of structures such as the HC, amygdala, prefrontal cortex, and midline thalamic nuclei [10]–[13], the VS is thought to utilize information of reward-predicting cues and contexts to guide goal-directed behavior [8],[14],[15]. This process is under strong control of the mesolimbic dopaminergic system, and its disruption has been associated with neuropsychiatric conditions such as drug addiction and obsessive-compulsive disorder [16]–[18]. Although the hippocampal formation projects directly to the VS, and this connection has been implicated in contextual conditioning [19], it is unknown how neural representations of contextual and motivational information are integrated and stored to enable the learning of place-reward associations.

In several brain areas, neuronal patterns evoked during behavior are reactivated during subsequent sleep [20]–[24]. Through modification of synaptic connections, this reactivation has been theorized to constitute an important step in memory consolidation [25]–[28]. Because hippocampal CA1 pyramidal cells exhibiting place fields during active behavior have been demonstrated to reactivate during sleep, it may be reasonably assumed that this replay pertains to spatial and contextual information [20],[21],[29],[30]. In contrast, reactivation in the VS is dominated by reward-related information [31]. Joint reactivation of HC and VS may enable the formation of a memory trace comprising both contextual and motivational components. In this study, we recorded activity from neuronal ensembles in the rat HC and VS simultaneously during wake and sleep episodes to examine whether the HC and VS reactivate coherently and to reveal the temporal dynamics of this process. First, during active behavior, much of the dynamics of hippocampal processing is governed by the theta rhythm, which has been hypothesized to function as a “read-in” or encoding mode for information acquisition and provides a means to temporally align spike sequences by way of theta phase precession [26],[32]–[34]. Therefore, we studied whether neural activity modulation by this rhythm in the awake state is correlated to reactivation during sleep. A second foremost question in this field, not yet addressed in previous multi-area recording studies [22],[24],[35], is whether cross-structural replay depends on the type of behavioral information coded by cell assemblies. To address this question, we investigated whether reactivation is preferentially associated with the expression of place fields and reward-related neural responses. Third, we planned to utilize joint HC-VS recordings to test a central tenet of theories of memory consolidation [25]–[28]. These theories posit that, after a learning experience, long-term episodic and declarative memories become gradually independent of hippocampal storage because this structure would repeatedly retrieve stored associative information over time and thereby orchestrate consolidation of memory traces in the neocortex and other target sites. A key point in these hypotheses is that replay is initiated and orchestrated by the HC, which prompted us to examine whether hippocampal activity leads the VS during reactivation.

## Results

**Figure 1:**
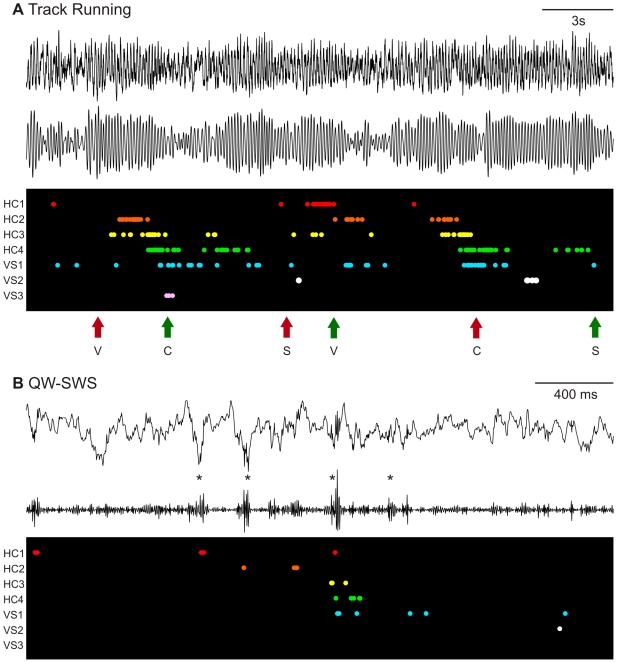
Sparse hippocampal and ventral striatal ensemble firing patterns during track running and postbehavioral rest. Example of the firing patterns of concurrently recorded hippocampal (HC1–HC4) and ventral striatal (VS1–VS3) cells during track running (A) and QW-SWS (B). Only cells that exhibited a place field or a reward-related correlate are shown in these graphs. Local field potentials recorded near the hippocampal fissure (A) and the hippocampal pyramidal cell layer (B) are plotted in parallel. (A) When the rat ran along the triangular track, the LFP displayed an oscillation of theta frequency (6–10 Hz, top: raw trace; bottom: filtered trace [6–10 Hz]). During the plotted period of 25 s, the rat encountered six reward sites (s = sucrose solution, v = vanilla desert, c = chocolate mousse) of which three contained a reward (green arrow) and the others were empty (red arrows). Each row in the black field represents one cell; its spikes are shown with colored dots. (B) During QW-SWS, the LFP displays large irregular activity intermitted with sharp wave-ripple complexes (top: raw trace; bottom: filtered trace [100–250 Hz]). Identified ripples are indicated with an asterisk (*). Several units that were activated during track running were reactivated within a short time period. Note the different time scales in (A and B). The relative firing order of pairs of HC and VS cells roughly corresponds to that observed during behavior (A).

Four rats were implanted with a tetrode drive allowing joint HC-VS recordings of spike trains of multiple neurons and local field potentials (LFPs) in each area. Daily recording sessions were composed of an episode of reward searching behavior flanked by two episodes of rest, which rats spent on a “nest” next to the track. The task was to run along a triangular track repeatedly and in one direction. On each lap, one of three reward wells was baited with a drop of one of three corresponding reward types; i.e., sucrose solution, vanilla desert, or chocolate mousse. An example of joint HC and VS ensemble recordings during track running and sleep is shown in Figure 1. First, we assessed reactivation of neuronal patterns using an explained variance (EV) method based on the spike correlations of cell pairs across all simultaneously recorded neurons [23],[36]. The EV reflects the extent to which the variance in the distribution of spike correlations during postbehavioral rest is statistically accounted for by the correlation pattern found during track running, factoring out the correlations present in prebehavioral rest. Joint HC-VS reactivation was examined during rest periods in which the rat was immobile, using only spike correlations between pairs composed of one HC and one VS neuron.

**Figure 2:**
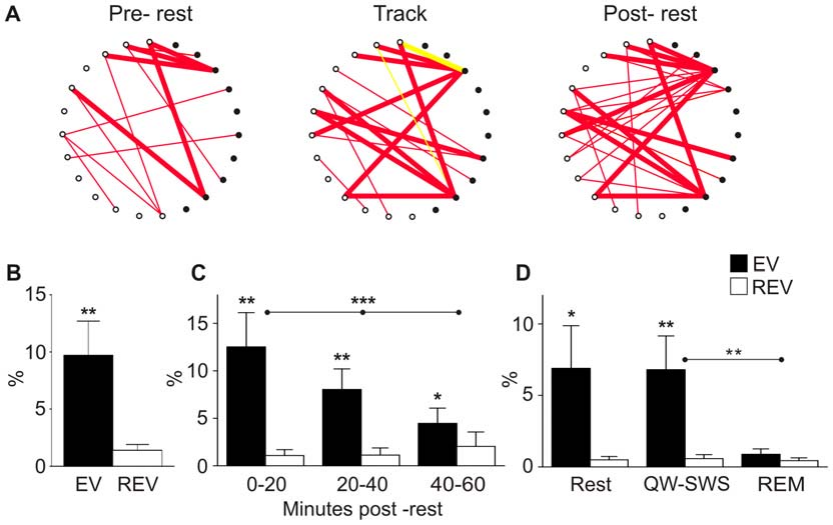
Coherent cross-structural reactivation in the hippocampal–ventral striatal circuitry. (A) Diagrams representing firing pattern correlations for pairs of simultaneously recorded hippocampal and ventral striatal units during periods of active behavior and rest in a single session. Individual neurons are represented as dots around the perimeter of a circle (filled dots: hippocampal CA1 units, *n* = 10; open dots: ventral striatal units, *n* = 13). Lines indicate a significant firing correlation between two neurons (red: positive, yellow: negative correlations). A pattern of correlations emerges during track running and is reinstated in postbehavioral rest, whereas it was largely absent in rest preceding behavior. (B) The EV was significantly larger than the control value (REV) when compared across sessions (**p<0.01). Error bars represent the standard error of the mean (SEM). (C) Temporal dynamics of joint reactivation were examined in three 20-min blocks of rest. Reactivation occurs at least up to an hour of rest after the experience but decays gradually (***p<0.002). (D) Reactivation observed during QW-SWS (**p<0.01) was not different from that found for the entire rest episodes (Rest, *p<0.05; *n* = 13 sessions). Reactivation was not observed in REM sleep. Between-condition statistics hold for EV values and the difference between [EV−REV].

We found coherent, cross-regional reactivation between ensembles of the HC and VS as expressed by an EV of 9.7±3.0%, which was significantly higher than the control measure, the reverse explained variance (REV: 1.4±0.5%, *p*<0.01, *n* = 21 sessions; Figure 2A and 2B; Figures S1 and S2; Table S1; Text S1). In the analyses conducted in this research, putative interneurons were excluded from the neuronal population, but it should be noted that including these interneurons yielded similar reactivation values (EV: 9.4±2.7%, REV: 1.9±0.7%; *p*<0.01). Analysis of the temporal dynamics of reactivation in 20-min blocks of concatenated rest revealed a gradually decaying reactivation which was significant for at least 1 h of postbehavioral rest (Figure 2C). Within periods of rest, reactivation was prominent especially during quiet wakefulness–slow-wave sleep episodes (QW-SWS; *n* = 13 sessions), but it was not significant for rapid eye movement (REM) sleep (Figure 2D). The lack of pattern recurrence during REM sleep was not attributable to its relatively short duration, undersampling of spikes, or its late occurrence after sleep onset compared to QW-SWS (Figure S3; Table S2; Text S1). Reactivation in the HC [36] and VS [31] occurs markedly during sharp wave-ripple complexes, i.e., short-lasting, high-frequency oscillations in the hippocampal LFP with associated bursts of large-scale neuronal firing that characterize QW-SWS [2],[37]. The same trend was observed for joint reactivation; however, the difference in reactivation values for time windows of 200 ms following ripple onset (“Ripples”) and during 200 ms following the onset of interripple intervals (“Intervals”) did not reach statistical significance, which most likely relates to the high variability across sessions (cf. [31]) (Ripples: EV: 5.9±2.6%, REV: 1.0±0.3%; Intervals: EV: 1.9±0.8%, REV: 0.5±0.2%; EV and between Ripples and Intervals [EV−REV]: n.s.; *n* = 14 sessions).

The existence of joint HC-VS reactivation raises the question of which physiological and behavioral factors are associated with the strength of this process. We examined three not mutually exclusive factors pertaining to (1) the modulation of the neural activity patterns by theta oscillations, (2) the correlation of neuronal firing patterns with behavioral parameters, and (3) the order in which neurons in different areas were activated. First, we computed the degree to which cells in each pair fired together, and then all of these correlation values per episode were pooled across sessions and animals. We next formed subgroups of cell pairs by partitioning the complete set of correlation values on the basis of the factor under scrutiny. Reactivation values were computed for these subgroups, and statistical significance was assessed by applying a bootstrapping procedure with resampling of pooled correlation values [22],[23].

The two-stage model of memory trace formation posits that theta oscillations are crucial for encoding information in the HC in the awake, active state [26]. The hippocampal theta rhythm may also have a role in governing the temporal organization of activity in target structures to ensure efficient communication [38],[39]. Thus, our first hypothesis holds that HC-VS reactivation will only be strong when information is cross-structurally aligned during encoding by a common temporal framing, the theta oscillation, creating windows of near-synchronous firing.

**Figure 3:**
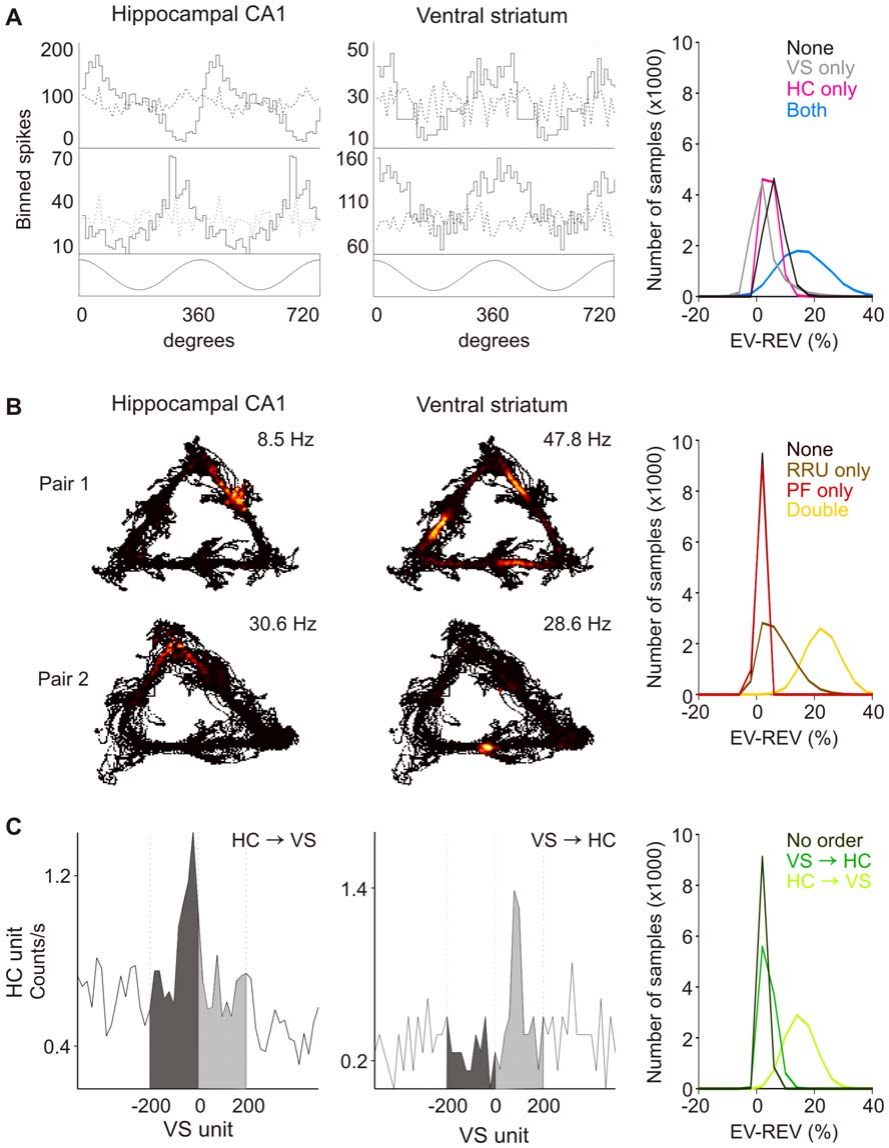
Reactivation of subgroups composed according to three firing pattern characteristics. (A) Modulation by theta oscillations. Four examples show binned spike counts (upper panels, solid lines) in relation to the hippocampal theta rhythm (bottom panels) for two successive theta cycles. Randomization of spike intervals abolished the relation between firing pattern and theta rhythm (dashed lines). Distributions of EV and REV values for each subgroup, obtained with bootstrapping, showed significant reactivation in all but the VS only subgroups. Reactivation in the Both Cells group was significantly stronger than in the other groups (*p*<1×10^4^; right-hand panel). (B) Processing of place and reward information. The left panel shows the spatial distribution of local firing rates of two hippocampal–ventral striatal pairs. The rat's trajectory is shown in black, and the firing rate of the neurons is color coded, ranging from low rates in dark red to their individual maxima (top right corners) in yellow and white. The Double Correlates group reactivated significantly, whereas the other subgroups did not. PF: place field; RRU, reward-related unit. (C) Firing order was defined by the difference in area between the light- and dark-shaded regions of cross-correlograms. Reactivation was observed in all groups, but the HC→VS group reactivated more strongly than the other groups.

**Figure 4:**
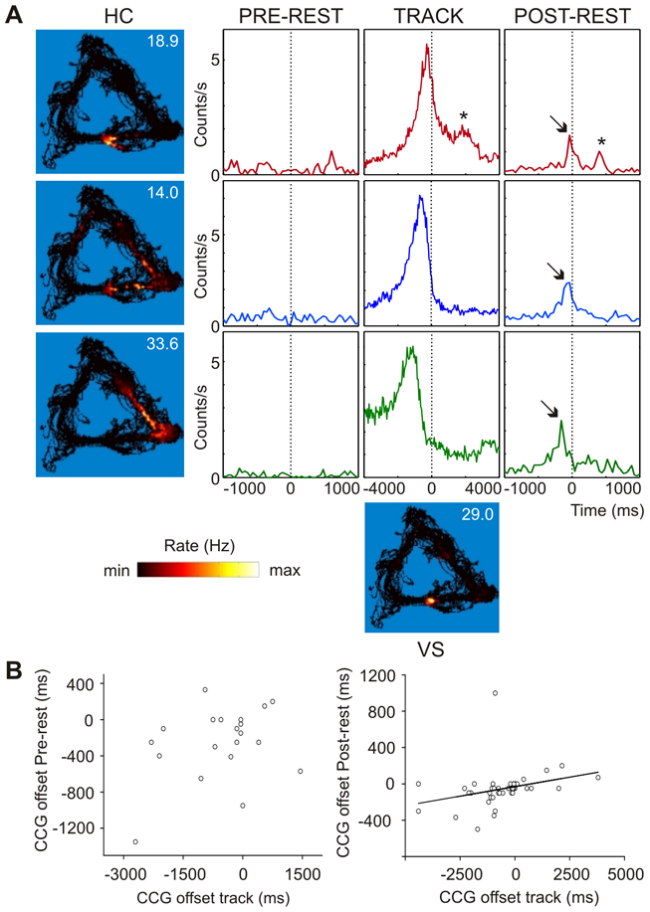
Order of firing is maintained in accelerated cross-structural replay. (A) Cross-correlograms for three pairs of simultaneously recorded neurons showing the temporal relation of firing during prebehavioral rest, track running, and postbehavioral rest. Hippocampal activity is synchronized on ventral striatal firing (time = 0, bin size 20 ms). Spatially distributed firing patterns of the neurons are shown in the blue squares (left: HC, bottom: VS; see also Figure 3B). During track running, the three pairs of neurons show correlated firing with peaks at different offsets relative to time zero. This correlated firing was absent in prebehavioral rest but reinstated during postbehavioral rest (indicated by arrows). In the topmost example, a secondary peak during track running is recurring in postbehavioral rest (indicated by the asterisk [*]). The relative time offsets of peaks were preserved from track running to postbehavioral rest. (B) Scatter plots of the temporal offsets of the peaks in the cross-correlograms (CCG) during track running and prebehavioral rest (left) and postbehavioral rest (right). The peak offsets during track running were correlated to the peak-offsets during postbehavioral rest (*R* ^2^ = 0.09, *p*<0.05), but not to prebehavioral rest. The peak offsets during postbehavioral rest were significantly reduced compared to track running, indicating accelerated reactivation. Note that cell pairs showing a significant peak in the cross-correlograms of track running and of at least *one* rest episode were included in analysis. Therefore, the number of data points is different in the left and the right panels.

During track running, robust theta oscillations were observed in the HC and VS. In both areas, cells were observed whose firing patterns were modulated by the local theta rhythm (*n* = 121 out of 263, 46.0%, in HC, and *n* = 20 out of 243, 8.2%, in VS (Figure 3A; FigureS4A). Ventral striatal units that were modulated by the local theta rhythm generally showed firing rate modulation also by hippocampal theta oscillations (*n* = 20, 8.2%). When the peak of the theta oscillation recorded near the hippocampal fissure was taken as synchronizing time point, CA1 cells fired at an average angle of 199.9±6.1° (range: 43.9°–356.4°) and VS cells fired at a slightly later phase (217.1±26.0°, range: 4.5°–336.3°; n.s.). Cell pairs were divided on the basis of modulation by the hippocampal theta rhythm, resulting in four subgroups: Both Cells (*n* = 140), HC only (*n* = 1,273), VS only (*n* = 81), and None Modulated (*n* = 1,422). Reactivation was observed for all but the VS only groups; its strength was significantly stronger in the Both Cells group compared to the None Modulated and HC only groups (Figure 3A; Table S3).

Our second hypothesis departed from the assumption that spike patterns are not reactivated equally, but are reprocessed especially when they convey behaviorally relevant information. For the HC-VS system, we predicted that cells expressing spatial (HC) and reward-related information (VS) should be preferentially reactivated. Location-specific firing was found for 102 out of 263 (38.8%) hippocampal cells. Place fields were distributed uniformly across the track; there was no indication that place fields occurred more frequently near reward sites or corners of the track. In contrast, a subset of VS neurons fired in close temporal relationship with reward site visits (41 out of 243 cells, 16.9%; Text S1). Reward-related responses were generally increments in firing rate and could be generated at one, two, or all three reward sites. Furthermore, they were often sensitive to either the presence or absence of reward. In line with previous studies on the VS [9],[31],[40], we will apply the term *reward-related* to all VS units showing significant responses time-locked to reward site visits.

Depending on the expression of place fields and reward-related correlates, cell pairs were grouped in four categories: Double Correlates (*n* = 192), Place Field only (*n* = 941), Reward-related Correlate only (*n* = 287), or No Correlates (*n* = 1496). The Double Correlates group showed very strong reactivation (EV: 22.9%, REV: 0.1%), whereas reactivation in the other three subgroups in this partition was not significant (Figure 3B; Table S3). Accordingly, the strength of coactivation of a place cell and a reward-related VS cell, expressed in the Pearson correlation coefficient, was positively correlated to the degree of spatial overlap of the firing fields on the track during task performance and postbehavioral sleep, but not during prebehavioral rest (prebehavioral rest: n.s.; track running: *R* ^2^ = 0.25, *p*<1×10^−12^, postbehavioral rest: *R* ^2^ = 0.03, *p*<0.02; *n* = 192).

A long-standing assumption in memory consolidation theory holds that the HC initiates and orchestrates reactivation in its projection areas [25]–[28]. This general process may be realized in several ways (see Text S1). In the HC-VS system, evidence suggests a particular variant of replay in which hippocampal ripples initiate reactivation locally and subsequently trigger a wave of enhanced excitability in the VS [23]. This variant implies that reactivation should be strong when a particular firing order is maintained: during replay, a hippocampal cell should fire predominantly in advance of a VS cell. During behavior, VS firing may also precede HC firing, but this order should not be associated with strong reactivation. The HC→VS order would also be consistent with the unidirectionality of the projection from HC to VS [13]. Despite the finding that sleep reactivation occurs in a “forward” direction, meaning that the order of firing during sleep is similar to the order during the preceding behavior [29],[30],[41], this critical assumption has yet to be confirmed or refuted. Hence, our third hypothesis holds that reactivation is strong when the information flow is organized according to a leading role of the HC.

The firing order of each cell pair was assessed by computing cross-correlograms [42],[43] and determining which order of firing was most prevalent using a “temporal bias” measure [29]. Three subgroups were distinguished, i.e., HC→VS pairs (*n* = 608), VS→HC pairs (*n* = 796), and No Clear Order, which included pairs that did not show a preferred firing order (*n* = 1,512). The HC→VS group reactivated strongly (EV: 15.2%, REV: 0.0%). Reactivation was also observed for the other groups, although the observed strengths were significantly lower than for the HC→VS group. (Figure 3C; Table S3).

Variations in reactivation measured across all of the subgroups partitioned according to each of the three factors could not be attributed to differences in varying numbers of cell pairs, differences in correlation strengths, or differences in spike counts (Text S1). Altogether, these results suggest that all three factors analyzed—modulation of both cells by the hippocampal theta rhythm, maintenance of the HC→VS firing order, and expression of a combination of a place field and a reward correlate—are associated with strong reactivation. However, since a reactivating cell pair may display multiple characteristics at the same time (e.g., behavioral correlates and a particular firing order; see Figure S4), we used a multilinear regression model to test whether the contribution of each cell pair to the session EV value was dependent on firing order, theta modulation, behavioral correlates, or any combination of these characteristics. First, the relative contribution of each cell pair to the session reactivation was estimated by excluding a pair from the simultaneously recorded population and recomputing the reactivation values. The difference between the session EV minus the EV after pair exclusion represents the estimated contribution of that pair to the session EV. Multilinear regression showed that both the expression of a double correlate and the HC→VS firing order were significant factors in explaining the contribution to the session EV (*p*<0.02 and *p*<0.002, respectively; theta modulation was not significant, *p* = 0.6). The combination of firing order and expression of a double correlate predicted the pair's contribution better than either one alone (*p*<0.0002). This analysis confirms the importance of the HC→VS firing order and expression of combined place and reward information during track running for subsequent reactivation and identified theta modulation as a less significant indicator.

We tested whether reactivation in the subgroup reactivating most strongly, i.e., the Double Correlates, was sparsely distributed as we previously showed for VS ensembles [31]. First, we assessed the contribution of each cell pair to the reactivation as explained above. To find an indication of how many cell pairs can be excluded to abolish reactivation, the pairs were sorted in descending order in terms of their contribution to the reactivation value [EV−REV]. Then the pairs were excluded one by one in a cumulative fashion starting with the highest contributor from the population, and reactivation values were computed each time a next pair was excluded. If the 17 (17/192, 8.9%) most contributing cell pairs were left out of the population, the [EV−REV] dropped below 5.0%. A total of 34 (17.7%) pairs could be removed before the [EV−REV] level decreased to 0.0%. This analysis indicates that, consistent with VS ensembles, reactivating cell pairs were also sparsely distributed in the HC-VS population.

We next explored whether HC-VS cell pairs fire in the same order during reactivation as during behavior and whether replay is accelerated relative to active behavior. For each pair of neurons that showed a place field and a reward-related correlate, we constructed three cross-correlograms, one for each task-episode (*n* = 192, Figure 4A). We compared the time offsets during active behavior and rest for pairs that showed significant peaks in the cross-correlograms of track running and in at least one of the rest episodes (*n* = 53, 27.6%). The time offsets of the peaks during track running were positively correlated to those in postbehavioral rest (*R* ^2^ = 0.09, *p*<0.05; *n* = 47), but not to those of prebehavioral rest (Figure 4B; *n* = 26). Thus, the recurrent firing patterns reflected the preceding experience. In this analysis, spatial overlap between the firing fields of a cell pair turned out not to be a prerequisite for concurrent firing during subsequent sleep, as 29.8% of the cell pairs that showed peaks in the cross-correlograms for task performance and postbehavioral rest exhibited nonoverlapping firing fields on the track. The peak offset in the cross-correlograms of track running ranged from −4.5 to 3.8 s and was significantly correlated to the spatial distance between the firing fields (*R* ^2^ = 0.27, *p*<0.001).

To determine whether the order of firing on the track was preserved or reversed in the subsequent rest episode the offset sign (+ or −) of the cross-correlogram peak relative to zero was considered. Peaks during track running and postbehavioral rest were consistently found with the same offset sign (43/47 = 89%, sign test, *p*<0.0001), which demonstrates that replay took place in a forward direction. In combination with the strong reactivation of cell pairs that exhibited the HC→VS firing order during track running observed in the subgroup-based reactivation analysis (Figure 3C), the preservation of firing order suggests that replay should be dominated by activity patterns in which HC firing largely precedes VS firing, both during track running and postbehavioral rest. Indeed, in the large majority of cell pairs that showed forward reactivation, the hippocampal cell fired preferentially before the striatal cell during both periods (36/43 = 83.7%), indicating that most of the reactivating cell pairs express a HC→VS order during behavior and sleep (sign test, *p*<0.0001). Thus, not only is the firing order preserved from the behavior to ensuing sleep, but apparently the HC also takes the lead in replay and the VS follows.

An additional analysis on all cell pairs with significant cross-correlogram peaks yielded similar results and confirmed that the preferential firing order during reactivation was not attributable to a lack of VS→HC correlations during track running (see Text S1). Like cross-structural reactivation, reactivation within hippocampal and ventral striatal ensembles also took place in a forward direction (see Text S1) (cf. [29] for HC).

Replay may occur at a different time scale than applicable during behavior [29],[30],[41]. We examined whether joint HC-VS firing patterns were replayed on an accelerated time scale. Peak times in the postbehavioral rest cross-correlogram occurred significantly closer to zero than during track running (track: −525.5±201.9 ms, postbehavioral rest: −53.2±28.5 ms; *p*<0.01; *n* = 47), showing an approximately10-fold compression (Figure 4). Replayed patterns appeared compressed and not merely truncated, because the shape of the cross-correlogram peaks with offsets of up to several seconds during behavior were re-expressed during sleep, including their offset from zero (Figure 4A).

## Discussion

Altogether, our results demonstrate coherent reactivation between the HC and a subcortical structure, and identify two major factors governing cross-structural reactivation in the HC-VS system, suggesting a plausible mechanism for consolidation of associative place-reward information. The first factor that significantly correlated to strong HC-VS reactivation bears on the dependence of reactivation on the coding of behaviorally relevant information. Cell pairs that exhibited a double correlate—one place field plus one reward-related correlate—showed the strongest reactivation among all four subgroups. In addition, the contribution to reactivation by individual pairs depended specifically on such a coexpression of behavioral correlates. The near-synchronous reiteration of spatial and motivational information during sleep may serve to integrate these types of information and support the learning of place-reward associations. Such associations are essential to predict and localize desired food and liquids within a known environment and are therefore fundamental to foraging behavior and learned behaviors such as conditioned place preference [5],[19],[44]. Like intra-area ventral striatal reactivation [31], cross-structural replay is dependent on a relatively small subset of cell pairs, indicating that it is a sparsely distributed phenomenon. If replay indeed supports memory consolidation, the formation of associations of a specific place-reward combination is likely to depend on a small minority of cells in the HC-VS circuitry.

The second significant factor in joint HC-VS replay is the preferred firing order of HC and VS cells, consistent across track running and subsequent sleep. The HC→VS firing order during track running was associated with a significantly elevated reactivation as compared to other temporal relationships, and the cell pair contributions to reactivation depended on this specific firing order. This organization of firing order obeys the direction of the anatomical projection [13],[45] and presents necessary, although not sufficient, evidence for a central tenet of consolidation theory, proposing the HC to initiate reactivation in its target structures, as predicted by Marr [25] and subsequent theorists [26]–[28] .

Our data provide several indications supporting that the observed cross-structural reactivation is the consequence of a coordinated process between the HC and the VS rather than of two separately, or coincidentally, reactivating ensembles. First, the temporal relationship between a pair of task-related hippocampal and ventral striatal cells was relatively consistent across task phases, resulting in significant peaks in the cross-correlograms of a substantial number of pairs both during track running and postbehavioral rest. If joint reactivation was just coincidental, the temporal firing relationship between cells in different structures is expected to be random, contrary to what was observed. Second, the timing of the peaks during track running and postbehavioral rest was correlated in the cross-correlogram analysis (Figure 4B), and furthermore, the time scale of sequential activation of firing patterns during postbehavioral rest was compressed compared to track running. Thus, temporal firing relations were consistent across different overall brain states (awake active versus SWS) and on accelerated time scales. Observing such results is very unlikely if the two structures would be reactivating without a systematic temporal relationship. Third, during behavior we identified pairs that fired in the order HC→VS and also in the order VS→HC. During reactivation in the postbehavioral rest, however, we found an overrepresentation of HC→VS pairs. If two ensembles reactivated independently, one would expect the ratio of HC→VS and VS→HC pairs to be similar during behavior and reactivation.

An important finding is that the joint reactivation is compressed by a factor of ten compared to the behavioral time scale of neuronal activation. Thus, at least several seconds of “real-time” joint place-reward information during behavior are brought together in a time frame of hundreds of milliseconds during sleep. This further supports the plausibility of a mechanism for the associative storage of place and reward information by way of synaptic weight changes in the HC-VS system. If a cell from the hippocampal formation, coding place, fires consistently and briefly in advance of a VS cell signaling reward (Figure 4), spike timing–dependent plasticity may be induced in their connection [46],[47].

Cross-correlogram analysis revealed that joint reactivation is not restricted to neuronal pairs that exhibit overlapping firing fields; peaks that were separated by up to about 4.5 s during behavior were found to recur during postbehavioral rest. In a scenario in which a series of place fields is followed by a reward-related correlate, this indicates that value information during SWS is not only paired with locations nearby, but also with more remote stages of the path leading to the reward site. Formation of reward-predicting representations should, by definition, obey the temporal order of predictor-reward events, a requirement that is met by the preferential HC→VS firing order during replay. In principle then, the characteristics of hippocampal-striatal replay are suitable for mediating the “backwards” association between reinforcements and cues and contexts situated progressively earlier in time. This temporally backwards referral is a key feature of conditioning theory and models of reinforcement learning [48]–[50].

Although the causal role of ensemble reactivation in memory consolidation remains to be proven, the temporally ordered cross-structural replay of spatial and motivational information during sleep illuminates a plausible offline mechanism by which information processed in different parts of the brain can be integrated to enable the composition and strengthening of memory traces comprising various attributes of a single learning experience.

## Materials and Methods

### Ethics Statement

All experimental procedures were in accordance with Dutch national guidelines on animal experimentation.

### Subjects

Four male Wistar rats (375–425 g; Harlan) were individually housed under a 12/12-h alternating light-dark cycle with light onset at 8:00 am. All experiments were conducted in the animal's inactive period. During training and recording periods, rats had access to water during a 2-h period following the experimental session, whereas food was available ad libitum. Rats were chronically implanted with a microdrive [51] containing five individually movable tetrodes directed to the dorsal hippocampal CA1 area (4.0 mm posterior and 2.5 mm lateral to bregma) and seven to the VS (1.8 mm anterior and 1.4 mm lateral to bregma) [52]. Reference electrodes were placed in the corpus callosum dorsal to the HC, and near the hippocampal fissure. A skull screw inserted in the caudal part of the parietal skull bone served as ground.

### Data Aquisition

Unit activity, local field potentials, and position data were acquired on a 64-channel Cheetah recording system (Neuralynx). Spike sorting was performed offline using custom cluster-cutting software as described in Text S1.

### Histology

Recordings of hippocampal CA1 neurons were made from 103 locations between 2.6 mm and 4.8 mm posterior and between 1.2 mm to 2.8 mm lateral to bregma compared to an atlas of the rat brain [52]. Ventral striatal tetrodes were situated between approximately 2.2 and 1.2 mm anterior to bregma and between 1.6 and 3.0 mm laterally. From a total of 140 recording sites, 58% was estimated to be situated in the core region and 42% in the shell region of the VS. Although most sessions were likely to contain recordings from both the core and shell region, six sessions were identified to contain core-only recordings. No gross differences were observed in the number, firing rate, or appearance of behavioral correlates that were estimated to be recorded from the core and the shell region. Moreover, cross-regional reactivation was observed for the core-only sessions, with EV and REV values similar to those observed for other sessions. Therefore, core and shell recordings were pooled.

### Resting State and Sleep Phase Identification

Pre- and postbehavioral rest episodes included all periods of at least 20 s in which the rat was in the flower pot and remained motionless; i.e., episodes of movement were excluded from analysis. Within these periods of rest, episodes of SWS were characterized by the presence of large irregular activity and the occurrence of sharp wave-ripple complexes in the LFP of the CA1 pyramidal layer [2],[37]. Ripples were detected each time the squared amplitude of the filtered LFP trace (100–300 Hz) crossed a threshold of 3.5 standard deviations (SD) for at least 25 ms. Because incidentally short periods of quiet wakefulness may have been included in SWS episodes, as these two phases share principal LFP characteristics, this state is referred to as quiet wakefulness–slow-wave sleep (QW-SWS). REM sleep periods were indicated by an elevated ratio (>0.4) of spectral density in the theta band (6–10 Hz) to the overall power of the LFP trace recorded near the hippocampal fissure. Their borders were refined upon visual inspection of the trace.

### Quantification of Reactivation

#### Session-based reactivation analysis

The assessment of covariation in firing rates and reactivation within an experimental session with the EV method was previously described [23],[36],[53],[54]. The temporal correlation between the firing patterns of two neurons was expressed in a Pearson's correlation coefficient that was computed for all concurrently recorded cell pairs using binned spike trains (50-ms bin size) of each rest/active episode. For assessment of cross-structural reactivation, pairs always consisted of one hippocampal and one ventral striatal cell; i.e., intra-area pairs were not taken into account. Separate analyses were conducted to examine intra-area reactivation; see Figure S2 and Text S1. All Pearson correlation coefficients of a particular episode (i.e., prebehavioral rest, track running, and postbehavioral rest) were assembled into a single matrix, and the similarity between the matrices was determined by computing a correlation coefficient for each of the three possible combinations of rest/active episodes. These matrix-based correlation values were used to assess which proportion of the variance in the postbehavioral correlation pattern can be explained by the pattern established during track running while controlling for any correlations that were present before the track running experience (i.e., in the prebehavioral rest). This quantity is expressed in the EV measure:
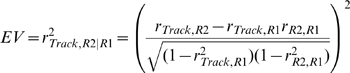
in which *R*1 and *R*2 represent the pre- and postbehavioral rest episodes, respectively, and for example, *r_Track_*_,*R*2_ equals the matrix correlation coefficient between the Track and Rest 2 pattern. EV equals the square of the partial correlation coefficient and is bounded between 0 and 1. The within-session control measure, i.e., REV, was derived by exchanging *R*1 and *R*2 in the previous equation, thereby switching the temporal order of episodes. EV and REV values were computed for all sessions that contained at least five well-isolated active neurons from each area and for 20-min time blocks of concatenated periods of quiet rest and sleep, i.e., periods of active behavior were excluded. Sessions that showed reactivation (EV>REV) in the first 20-min rest block after track running were used to assess decay of reactivation (*n* = 15). Sleep phase–dependent reactivation was computed for sessions that showed more than 4 min of REM and QW-SWS in each rest episode (*n* = 13). Differences between EV and REV values were tested for statistical significance with the Wilcoxon matched-pairs signed rank test.

#### Reactivation analysis for subgroups

For assessing reactivation in subgroups of cell pairs (e.g., “Both” and “None” modulated in the section on theta modulation), all Pearson correlation coefficients for cell pairs within each subgroup were pooled across all sessions and all rats before the EV and REV values were computed. Estimates of the mean and variance of these values were derived using a bootstrapping procedure in which random samples were taken (*n* = 10,000) from the observed set of Pearson correlation coefficients [22],[55]. During resampling, the triplets of correlation coefficients belonging to the three rest/active episodes were kept together, and the created samples were of the same size as the original subset. The resampling procedure included replacement, thus samples may have contained multiple copies of one triplet, whereas others were omitted. For each sample, reactivation measures were computed resulting in distributions of EV and REV and the difference [EV−REV] values for each subset. After bootstrapping, 95% confidence intervals were determined for the [EV−REV] distribution. A subgroup was considered to reactivate when the 2.5 percentile of this distribution exceeded zero. Differences in reactivation strength between two subgroups were statistically assessed using the [EV−REV] values in Mann-Whitney *U* test (MWU; *p*<1×10^−4^).

### Theta-Modulated Firing

Modulation of a cell's firing pattern to the theta oscillation was determined by first filtering LFP traces recorded from the hippocampal fissure and the VS using a Chebyshev type-1 bandpass filter between 6 and 10 Hz. Binned spikes (10°/bin) were then plotted relative to the theta peaks of two successive theta periods. The spike distribution was considered nonuniform when the Rayleigh score was <1×10^−5^. The phase angle of the spikes was determined by computing the Hilbert transform of the filtered theta signal. Firing of a unit was considered as being modulated by the theta rhythm when shuffling of the spikes abolished the nonuniformity of the spike firing distribution as assessed with the Rayleigh score.

### Identification of Place Fields

To characterize spatially selective firing fields, instantaneous firing rates were computed for bins of 50 ms. The spatial position of the rat's head was determined by creating a one-dimensional representation of the track and using a resolution of 2.3 cm. Mutual information was computed between the binned spike trains and the position, and corrected for finite sampling size [56],[57]. A cell was considered to express a place field if its firing rate during track running was at least 0.3 Hz and if it carried at least 0.25 bits/spike of spatial information.

### Identification of Reward-Related Firing Patterns

Peri-event time histograms were constructed for the rewarded and nonrewarded condition for each reward site and were synchronized on crossings of offline installed “virtual photobeams” positioned at the points where the rat was just reaching the reward sites. Reward-related responses were assessed within a period of 1 s before and 1 s after arrival at a reward site, using a bin resolution of 250 ms. Spike counts in the eight bins comprising the reward period were each compared to three separate control bins taken from the corner passage opposite to the well under scrutiny within the same lap. A bin of the reward period was only considered significantly different when the Wilcoxon matched-pairs signed rank test indicated significance from each of the three control bins (*p*<0.01). A reward-related response comprised one or more bins that were significantly different from control bins. Control bins did not show marked deviations from overall baseline firing as checked in peri-event time histograms and plots of the spatial distribution of firing rates. Differences between the responses at different reward sites were assessed with a Kruskal-Wallis test (*p*<0.05) followed by a MWU (*p*<0.05), whereas rewarded versus nonrewarded conditions were compared using MWU (*p*<0.05).

### Cross-Correlograms and Temporal Bias Method

Cross-correlograms were constructed according to Perkel et al. [58] and Eggermont [43]. Spikes were binned into 10- or 50-ms intervals, and the cross-correlation was examined across at least three time windows; viz. [−500, 500] ms, [−2,000, 2,000] ms and [−5,000, 5,000] ms. The firing order of pairs of hippocampal and striatal cells was assessed with the temporal bias method [29] using cross-correlograms with the spikes of the striatal cell serving as reference. The ordinate expressed spike counts per second, which was integrated across intervals of 200 ms before (I) and after (II) zero. The difference between II minus I divided by the sum of the spike counts determined the temporal bias score. If this score was negative, the HC was determined to fire preferentially before the striatal cell. If this score was positive, the preferred firing order was in the opposite direction. The classification No Clear Order was assigned when the scores (I and II) were approximately equal or when the cross-correlogram did not have a clear single maximum.

To estimate the significance of peaks in the cross-correlograms, the mean expected number of joint spike counts μ and the levels of μ±3 SD (corresponding to *p* = 0.0013) were computed to provide indications for nonrandom excursions of spike counts above or below the expected range [43]. Each cross-correlogram was then subjected five times to a spike-shuffling subtraction procedure [42],[58]. Peaks were accepted as significant only when they exceeded the +3 SD threshold above the mean in each of the five repetitions.

## Supporting Information

Figure S1 Cross-structural reactivation is cell and time specific.
(0.05 MB PDF)

Figure S2 Cross-structural and intra-area reactivation.
(0.04 MB PDF)

Figure S3 Lack of REM sleep reactivation is not attributable to undersampling or decay.
(0.05 MB PDF)

Figure S4 Distributions of firing properties during track running across hippocampal and ventral striatal units.
(0.35 MB PDF)

Table S1 Numbers of sessions, cells, and cell pairs recorded per rat.
(0.02 MB PDF)

Table S2 Mean spike counts per cell during REM sleep and QW-SWS segments.
(0.02 MB PDF)

Table S3 Reactivation values of subgroups composed on the basis of three physiological and behavioral factors.
(0.07 MB PDF)

Text S1 Supporting materials and methods, results, and discussion.
(0.09 MB PDF)

## Abbreviations

EV: explained variance
HC: hippocampus
LFP: local field potential
QW-SWS: quiet wakefulness–slow-wave sleep
REM: rapid eye movement
REV: reversed explained variance
SWS: slow-wave sleep
VS: ventral striatum
