# Supplementary material for: Hippocampus Leads Ventral Striatum in Replay of Place-Reward Information
Source: PLoS Biol. 2009 Aug 18;7(8):e1000173. doi: 10.1371/journal.pbio.1000173 (PMC2717326; doi:10.1371/journal.pbio.1000173)
Supplement: Figure S1 — Cross-structural reactivation is cell and time specific. (0.05 MB PDF) [file pbio.1000173.s001.pdf]

Lansink et al., Figure S1

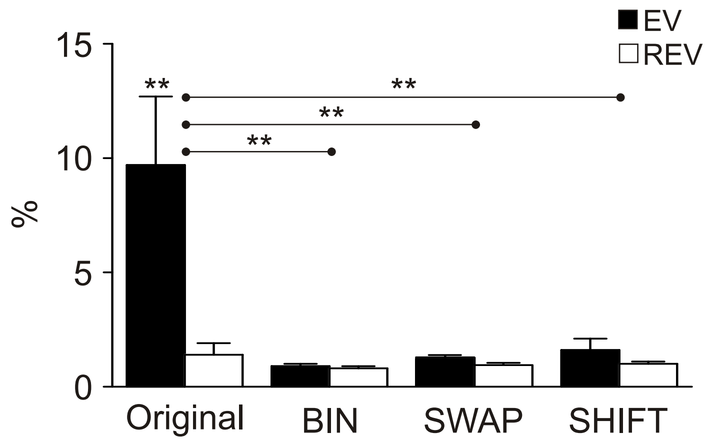

Figure S1: Cross- structural reactivation is cell- and time specific. Cross-structural reactivation was observed across 21 sessions (Original: Wilcoxon´s matched-pairs signed rank test, **$p < 0.01$). To test whether the reactivation was cell- and time specific, spike train vectors of the track running episode were randomized according to three different protocols. Shuffling of the time bins within the spike train vector of each cell (BIN), exchanging cell identities (SWAP) and disruption of the temporal alignment of the spike train vectors (SHIFT) each abolished reactivation. Residual EV and the difference (EV-REV) after randomization were significantly reduced compared to the original values. Error bars indicate SEM.
