# Supplementary material for: Hippocampus Leads Ventral Striatum in Replay of Place-Reward Information
Source: PLoS Biol. 2009 Aug 18;7(8):e1000173. doi: 10.1371/journal.pbio.1000173 (PMC2717326; doi:10.1371/journal.pbio.1000173)
Supplement: Figure S2 — Cross-structural and intra-area reactivation. (0.04 MB PDF) [file pbio.1000173.s002.pdf]

Lansink et al., Figure S2

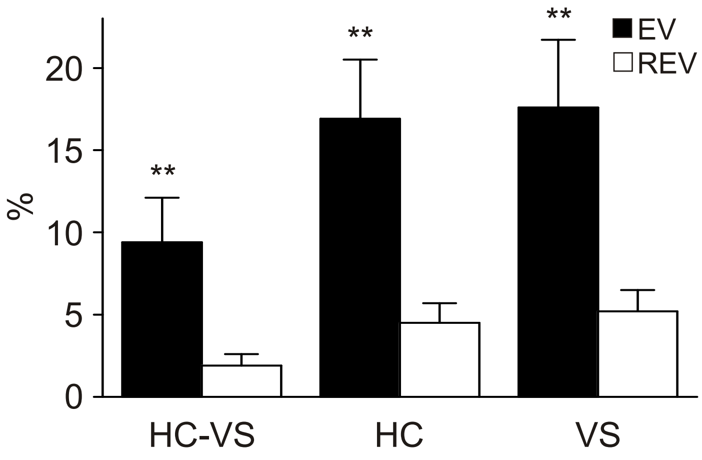

Figure S2: Cross-structural and intra-area reactivation. Besides cross-structurally (HC-VS; **$p < 0.01$), reactivation also occurred within the hippocampus (HC) and the ventral striatum (VS**)**. Note that for the assessment of cross-structural reactivation only cell pairs that consisted of one hippocampal and one ventral striatal unit were taken into account whereas intra-area reactivation analysis included cell pairs of which both members were recorded from the same area. Error bars indicate SEM.
