# Supplementary material for: Hippocampus Leads Ventral Striatum in Replay of Place-Reward Information
Source: PLoS Biol. 2009 Aug 18;7(8):e1000173. doi: 10.1371/journal.pbio.1000173 (PMC2717326; doi:10.1371/journal.pbio.1000173)
Supplement: Figure S3 — Lack of REM sleep reactivation is not attributable to undersampling or decay. (0.05 MB PDF) [file pbio.1000173.s003.pdf]

Lansink et al., Figure S3

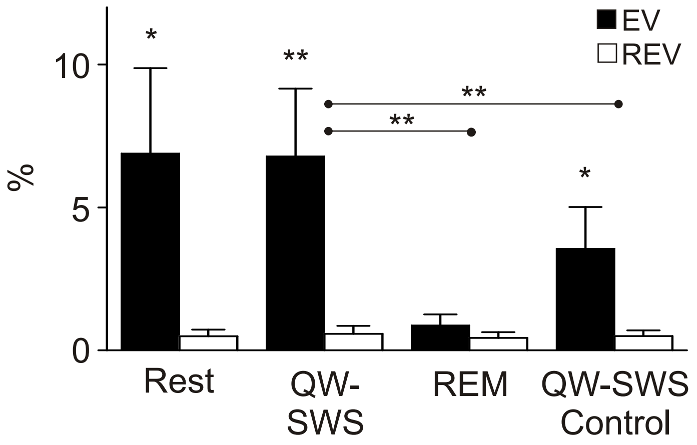


**Figure S3: Lack of REM sleep reactivation is not attributable to undersampling or decay.** Reactivation occurred during periods of QW-SWS (** p<0.01) but was absent in REM sleep. The lack of reactivation in REM sleep could not be explained by the total REM sleep time or to the relatively late occurrence of REM sleep periods after sleep onset, because post-REM segments of QW-SWS showed significant reactivation (QW-SWS control, *p<0.05). Reactivation in the overall QW-SWS phase was significantly larger than in REM sleep or post-REM sleep segments of QW-SWS (** p<0.01). Error bars indicate SEM.
