# Supplementary material for: Hippocampus Leads Ventral Striatum in Replay of Place-Reward Information
Source: PLoS Biol. 2009 Aug 18;7(8):e1000173. doi: 10.1371/journal.pbio.1000173 (PMC2717326; doi:10.1371/journal.pbio.1000173)
Supplement: Figure S4 — Distributions of firing properties during track running across hippocampal and ventral striatal units. (0.35 MB PDF) [file pbio.1000173.s004.pdf]

Lansink et al., Figure S4

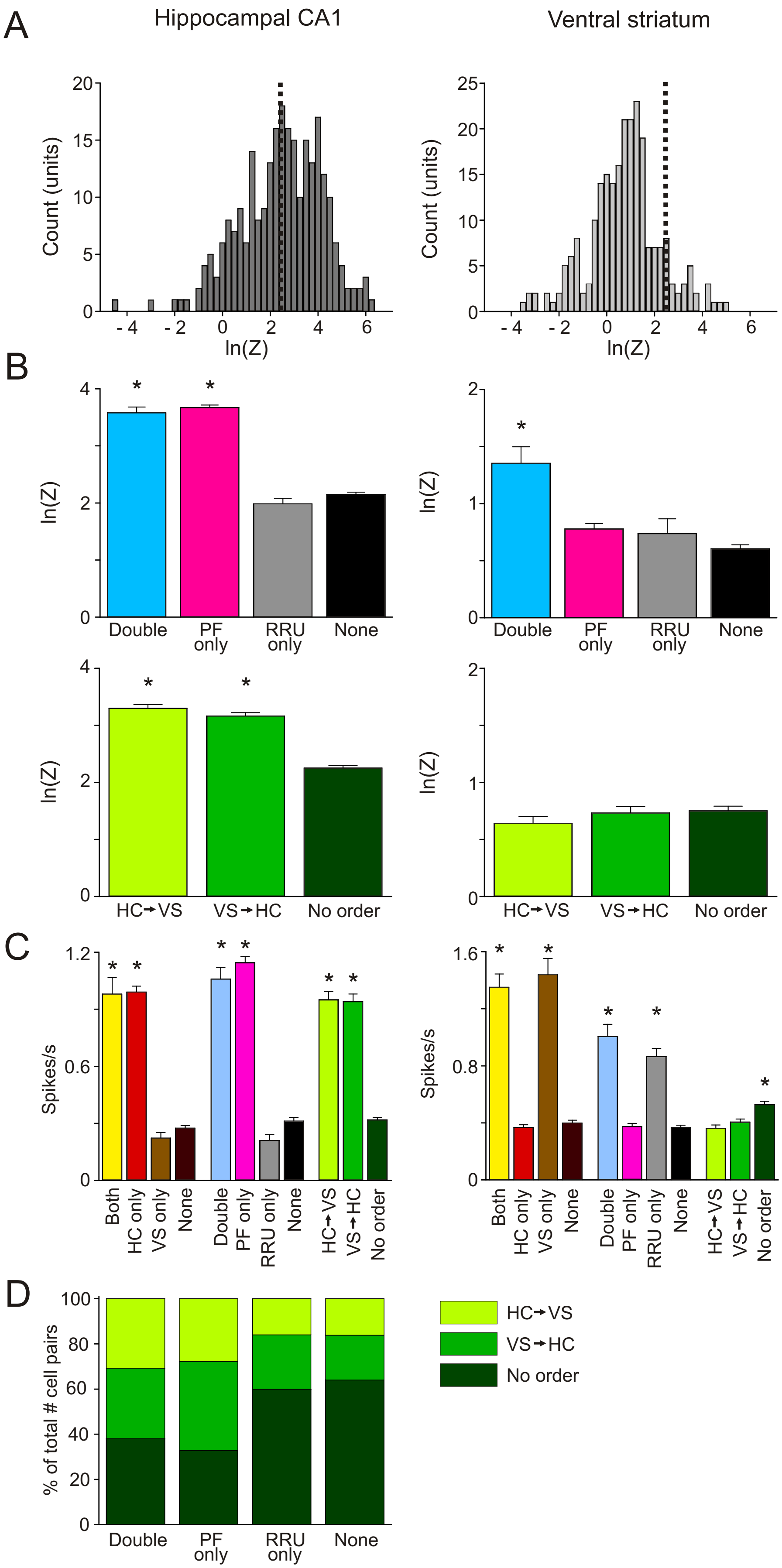

Figure S4: Distributions of firing properties during track running across hippocampal and ventral striatal units. A: The degree to which the firing of hippocampal (left) and ventral striatal (right) units during track running was modulated by theta oscillations recorded from the hippocampal fissure is expressed in distributions of log transformed Rayleigh's Z values. Probability of significance associated with Z is $p \approx e^{-Z}$ and therefore $\ln(Z) = \ln(-\ln(p))$. Dashed lines ($Ln(Z) = 2.44$) indicate a significance level of $p = 1 \cdot 10^{-5}$. Note that the logarithmic transformation of Z values introduces a double logarithmic scale of the abscissa so that Z values of [1,2,3,4] correspond to p levels of approximately [10^{-1}, 10^{-3}, 10^{-9}, 10^{-24}] *(Siapas et al. (2005) Neuron 46:141-151)*. B: Mean degree of theta modulation for cells belonging to the subgroups in the partitions 'Expression of behavioral correlates' and 'Firing order'. The left and right columns display firing properties of HC and VS members of the partition under scrutiny, respectively. The groups that showed the highest or near highest mean ln(Z) value and that were also significantly different from other subgroups are indicated with an asterisk (*; Mann-Whitney's U-test (MWU), HC units: $p < 1 \cdot 10^{-3}$, VS units $p < 0.01$). Note that both HC and VS members of 'Double Correlates' pairs showed relatively strong theta modulation. Strong theta modulation was also found for HC members of both the 'HC first' and 'VS first' groups C: Mean firing rate during track running for each subgroup in the three partitions. The groups within each partition that showed the highest firing rates and that were also significantly different from other subgroups are indicated with an asterisk (*; MWU, HC units: $p < 1 \cdot 10^{-3}$, VS units $p < 1 \cdot 10^{-5}$). The mean firing rate of the subgroups was not correlated to the value of reactivation measures for hippocampal and ventral striatal cells. D: Proportions of cells in the subgroups of the 'Expression of behavioral correlates' partition that maintained a specific firing order. Error bars indicate SEM.
