# Supplementary material for: Hippocampus Leads Ventral Striatum in Replay of Place-Reward Information
Source: PLoS Biol. 2009 Aug 18;7(8):e1000173. doi: 10.1371/journal.pbio.1000173 (PMC2717326; doi:10.1371/journal.pbio.1000173)
Supplement: Table S1 — Numbers of sessions, cells, and cell pairs recorded per rat. (0.02 MB PDF) [file pbio.1000173.s005.pdf]

Lansink et al.

Table S1: Summary of sessions, cells and cell pairs recorded per rat

	Number of sessions	Number of hippocampal cells	Number of ventral striatal cells	Number of HC-VS cell pairs
Rat 1	2	19	39	368
Rat 2	6	82	55	699
Rat 3	6	69	65	710
Rat 4	7	93	84	1139
total	21	263	243	2616
