# Supplementary material for: Hippocampus Leads Ventral Striatum in Replay of Place-Reward Information
Source: PLoS Biol. 2009 Aug 18;7(8):e1000173. doi: 10.1371/journal.pbio.1000173 (PMC2717326; doi:10.1371/journal.pbio.1000173)
Supplement: Table S2 — Mean spike counts per cell during REM sleep and QW-SWS segments. (0.02 MB PDF) [file pbio.1000173.s006.pdf]

Lansink et al.
Table S2: Mean spike counts per cell during REM sleep and QW-SWS segments

	REM sleep	*QW-SWS control segments*
Hippocampal CA1		
pre-behavioral rest	199.1 ± 42.1	252.6 ± 70.3
post-behavioral rest	231.9 ± 40.4	305.9 ± 71.5*
Ventral Striatum		
pre-behavioral rest	179.8 ± 24.6	102.7 ± 16.8**
post-behavioral rest	232.6 ± 32.9	145.1 ± 19.7**

Mean spike counts per cell were computed for episodes of REM sleep and for QW-SWS control segments, i.e. periods of QW-SWS that were of identical total length compared to REM sleep but occurred at more remote times after sleep onset (* $p< 0.05$; ** $p<0.001$).
