# Supplementary material for: Hippocampus Leads Ventral Striatum in Replay of Place-Reward Information
Source: PLoS Biol. 2009 Aug 18;7(8):e1000173. doi: 10.1371/journal.pbio.1000173 (PMC2717326; doi:10.1371/journal.pbio.1000173)
Supplement: Table S3 — Reactivation values of subgroups composed on the basis of three physiological and behavioral factors. (0.07 MB PDF) [file pbio.1000173.s007.pdf]

Lansink et al.

Table S3: Reactivation values of subgroups composed on the basis of three physiological and behavioral factors.

	Cell pairs (n)	Sessions (n)	EV (%)	REV (%)	EV-REV (%)
Firing modulated by HC theta rhythm					
Both Cells	140	11	17.3*	0.0	17.3 ‡ †
HC only	1273	20	4.5*	0.0	4.5 ‡ §
VS only	81	11	1.6	0.0	1.6
None Modulated	1422	21	6.5*	0.1	6.4 † §
Expression of behavioral correlates					
Double Correlates	192	16	22.9*	0.1	22.8
Place Field only	941	21	0.9	0.0	0.9
Reward related Correlate only	287	16	8.9	1.3	7.6
No Correlates	1496	21	0.5	0.1	0.4
Order of firing					
HC → VS	608	21	15.2*	0.0	15.2 ‡ †
VS → HC	796	21	4.2*	0.2	4.0 ‡ §
No Clear Order	1512	21	2.1*	0.0	2.1 † §

Occurrence of reactivation was statistically assessed by considering whether the 2.5 percentile of the difference (EV-REV) distribution of each subgroup exceeded 0 (*). The difference in reactivation between subgroups of the same partition was tested for statistical significance with Mann-Whitney's U-test using the difference (EV-REV) as measure for reactivation, but EV values yielded very similar results ($p < 1 \cdot 10^{-4}$; ‡ † § indicate significant differences between reactivating groups within each partition).
