# Supplementary material for: Hippocampus Leads Ventral Striatum in Replay of Place-Reward Information
Source: PLoS Biol. 2009 Aug 18;7(8):e1000173. doi: 10.1371/journal.pbio.1000173 (PMC2717326; doi:10.1371/journal.pbio.1000173)
Supplement: Text S1 — Supporting materials and methods, results, and discussion. (0.09 MB PDF) [file pbio.1000173.s008.pdf]

Text S1

Hippocampus Leads Ventral Striatum in Replay of Place - Reward Information

Carien S Lansink[1], Pieter M Goltstein[1], Jan V Lankelma[1], Bruce L McNaughton[2] and Cyriel MA Pennartz[1].

[1] Swammerdam Institute for Life Sciences – Center for Neuroscience, University of Amsterdam, Amsterdam, the Netherlands. [2] Canadian Center for Behavioral Neuroscience, The University of Lethbridge, Lethbridge, Canada

Supporting Materials and Methods

Behavioral paradigm Prior to surgery, rats learned to shuttle back and forth on a linear track (185 cm long x 10 cm wide, 40 cm elevated from the floor). Rewards (sucrose solution, 10%, vanilla desert or chocolate mousse) were provided on the track ends according to a partial reinforcement schedule. Recording sessions consisted of a rest period (pre-behavioral rest, 20-60 min) followed by a phase of reward searching behavior on a triangular track (20 min) and concluded with a second period of rest (post-behavioral rest, 60-120 min). All rats were unfamiliar with this track when recording session commenced. Rats were required to run along the track repeatedly and in one direction stopping only at reward wells positioned in the center of each arm to check for the presence of a reward. Each type of reward mentioned above was assigned to one of the three reward wells and the reward-location combination remained fixed throughout the recordings. Each lap, one reward was delivered to its corresponding cup according to a pseudorandom schedule. Rats rested on a towel in a wide flowerpot located next to the track.

Data acquisition Recordings were conducted using a 64 channel Cheetah recording system (Neuralynx, Bozeman, MT, USA). Activity of individual neurons was sampled during one ms windows (32 kHz, amplifier gain 5000, filter settings: 600-6000 Hz), whenever the voltage signal exceeded a manually preset voltage threshold. Local field potentials (LFPs) were continuously

sampled at 1690 Hz and band-pass filtered between 1 and 475 Hz. The rat’s headstage was equipped with an array of light-emitting diodes which allowed to track the position of the rat and to indicate body movements during periods of rest (60 frames/seconds, resolution ~2.5 mm/pixel).

Spike Sorting Groups of spikes belonging to a single unit were discriminated from other clusters and noise events on the same tetrode on the basis of waveform properties across the four channels of a tetrode using standard automated and manual clustering methods (Bubbleclust by P. Lipa, University of Arizona, AZ, Tucson U.S.A. and MClust by A.D. Redish, University of Minnesota, Minneapolis, MN, U.S.A. respectively). Clusters that were selected for analysis exhibited less than 0.1% of spike intervals within a 2 ms period in their inter-spike interval histograms and emitted at least 20 spikes in each task episode. Putative interneurons were discriminated from principal cells on the basis of average firing rate (>8 Hz) and waveform characteristics such as small peak to valley width and the valley shape and were not included in the analysis. See Table S1 for details on the number of sessions, neurons and cell pairs recorded for each rat.

Reactivation of neuronal sequences In addition to cross-correlograms, a ranked-order sequence analysis of multi-neuron spike trains has been applied to hippocampal and neocortical datasets to demonstrate temporally structured replay [1-3]. Typically, templates consisting of ranked-order sequences are extracted from the period of active behavior, while sequential replay is indicated when a partial or complete ‘match’ is found between the template and a multi-neuron spike sequence in a putative reactivation period, be it in an awake or sleeping state. Although this method appears to work well for hippocampal datasets, it is considered less suitable for studying HC-VS replay in the current task, first because reward-related neurons in the VS often showed multiple foci of intense firing on the behavioral track, which does not result in an unambigous sequence when ordered together with firing of hippocampal place cells. Second, the probabilistic reward-search task we applied did not result in strong behavioral regularity, because in some trials a reward was present at a given site but not in other trials. If a VS cell was responsive to

reward at that same site, it fired in close relation to activity of an hippocampal cell having a nearby place field in some trials but not others, again leading to variability in the elicited spike sequences. Because correctly replayed multi-neuron spike sequences appear to be quite sparse even under conditions of strong behavioral regularity [1-3], they are probably very hard to detect against a background of other, similar sequences arising from differently structured trials within the same session. In contrast, cross-correlograms provide a lumped measure of temporally related firing of two cells and may therefore be less sensitive to variations in behavior and concomitant VS firing.

Regression analysis Multi-linear regression was used to determine whether the contribution of the cell pairs to the Explained Variance per session was dependent on firing characteristics of the neuronal pairs; i.e. modulation by the theta oscillation, expression of place and/or reward-related firing patterns, order of firing and any combination of these factors. Each partition was used as regressor and the cell pairs were assigned a value according to the subgroup they belonged to (e.g. 'Double Correlates': 4, 'Place Field only': 3, 'Reward-related Correlate': 2 and 'No Correlates': 1). Thus the values of the regressors varied between 1 and the number of subgroups in the partition. Similar results were obtained when a value was assigned to the number of correlates that a pair expressed (e.g. 'Double Correlates': 2: 'Place Field only' and 'Reward-related Correlate only': 1 and 'No Correlates': 0).

Histology Following termination of an experiment, the tetrode endpoints were marked by a small lesion resulting from passing a 25 μA current for 10 s through one of the leads of each channel. The next day, rats were transcardially perfused with 0.9% saline solution followed by 4.0% paraformaldehyde in phosphate-buffered saline (0.1 M, pH 7.4). Coronal brain sections (40 μm) were cut on a Vibratome and Nissl-stained for verification of tetrode tracks and endpoints.

Supporting Results

Behavior on the triangle track On the track, rats ran in one direction and paused shortly at each reward site to check for reward availability. As the rats gained experience on the track, the average number of laps completed in one session increased (15.3 ± 4.1 in the first session to 62.3 ± 6.2 in the tenth, linear regression R^2 = 0.53, p<0.0001). The time between two reward site visits was significantly longer when the rat consumed a reward than when the well was not baited (16.54 ± 0.38 and 7.65 ± 0.25 s, Wilcoxon´s matched-pairs signed rank test, p<0.0001). The travel-time between wells did not decrease when a few consecutive wells were empty.

Reactivation strength of individual sessions expressed as the difference between (EV-REV) was highly variable but on average increased across sessions and was correlated to the progression through the sessions (linear regression R^2 = 0.20, p<0.05) and the number of laps completed on the track (R^2 = 0.46, p<0.001). These positive correlations do not confirm or contradict a role for reactivation in learning and memory consolidation per se. The increasing strength of reactivation with experience on the track might reflect a learning process underlying improving task performance. Alternatively, the same result may be explained by an enhancement of reactivation detectability along with task familiarity [4]. As the behavior of the rats becomes more regular and repetitive with training, neuronal patterns may well be more consistently repeated.

Quality of cluster isolation is not related to strength of HC-VS reactivation

Previous studies have reported that the quality of cluster isolation may have profound consequences for the detection of correlated firing [5]. To ensure that the cross-regional reactivation observed in this study does not depend on differences in cluster isolation, the cluster quality measures L-ratio and Isolation Distance [6] were computed for each cell recorded. Next, we assessed whether a correlation existed between the strength of reactivation (EV and (EV-REV)) and the cluster isolation quality of both hippocampal and striatal units in the session-based condition and in the subgroup-based condition (i.e. ‘Both Modulated’, ‘Double Correlate’, etc).

The strength of session-based reactivation (EV-REV)) was not significantly correlated to the session medians of LRatio, log(LRatio) and Isolation Distance for hippocampus or ventral striatum. Likewise, the reactivation strength (EV-REV) of the different subgroups was not correlated to any of the cluster isolation measures of hippocampal and ventral striatal neurons. Thus, within the sample of well-isolated units, session-based HC-VS reactivation was not dependent on cluster isolation quality and reactivation differences between subgroups could not be attributed to varying isolation quality.

Randomized controls for verification of cross-structural reactivation To examine whether the observed cross-structural reactivation is attributable to cell- and time-specific firing correlations or could have arisen by chance alone, the significance of the observed EV and REV values was assessed by comparing these to reactivation values obtained after randomization of the binned spike trains. Three different randomization procedures were applied on the binned spike trains of the track running period [7]. First, to control for the possibility that consistent differences in firing rate accounted for the observed reactivation, the temporal order of spike trains was disrupted within each cell while the spike numbers were kept constant. In this *BIN* condition, time bins were randomly exchanged within the spike train vector of each cell. Secondly, to ensure that the observed reactivation is not due to a broad modulation of firing patterns by vigilance state, the spike train vectors were randomly reassigned to different cells (*SWAP* condition). Third, to test whether reactivation depends on the temporal alignment of the spike trains of cell pairs, entire spike train vectors were individually shifted across a random time interval with a maximum of 10 s forward or backwards (*SHIFT* condition). The shift was circular so that bins that were removed from one end of the vector were reinserted at the other end. In all three control conditions, the randomization procedures reduced the difference between EV and REV values to insignificant levels (Figure S1; BIN: EV: 0.9 ± 0.1%, REV 0.8 ± 0.1%; SWAP: EV: 1.2 ± 0.1%, REV 0.9 ± 0.1 %; SHIFT: EV: 1.6 ± 0.5 %, REV: 1.0 ± 0.1%; Wilcoxon´s matched-pairs signed rank test, n.s.). Furthermore, in all conditions, reactivation measures were significantly decreased compared to the reactivation observed with the original spike train vectors

($p < 0.01$). The absence of reactivation in the randomized control conditions indicates that the reactivation observed originally depends on the specific spike timing relationships between HC and VS cells.

Control procedure verifying absence of reactivation in REM sleep In pre- and post behavioral rest episodes, rats spent significantly more time in QW-SWS than in REM sleep (pre-QW-SWS: 19.3 ± 2.4 min, pre-REM sleep: 9.0 ± 0.8 min, post-QW-SWS 37.0 ± 2.9 min, post-REM: 11.6 ±1.0 min, $p<0.01$). Compared to quiet wakefulness-slow wave sleep, episodes of REM sleep were short and occurred at relatively remote times after sleep onset. In combination with a decaying reactivation, these factors may provide an explanation for the absence of reactivation during REM sleep. To examine this, reactivation was computed over segments of quiet wakefulness-slow wave sleep that were of identical length as the REM epochs but occurred at later time points in the rest period. Significant reactivation was observed in these quiet wakefulness-slow wave sleep segments (EV: 3.6 ± 1.4%, REV: 0.5 ± 0.2%; $p< 0.05$; Figure S3), indicating that the lack of pattern recurrence during REM sleep is most likely unrelated to its short duration and its late occurrence after sleep onset.

Hippocampal cells emitted on average significantly more spikes per cell during the QW-SWS segments than in REM sleep during the post- but not the pre-behavioral rest phase (Table S2, *$p<0.05$). In contrast, striatal cells were more active during REM sleep episodes than during the SWS sleep segments in both rest phases (**$p<0.001$).

Behavioral correlates of ventral striatal firing patterns Out of 243 recorded ventral striatal units, 41 (16.9%) showed significant firing rate changes in close association with reward site arrivals. Although these changes were by and large firing rate increments (40, 97.6%), response patterns were heterogeneous. Responses were found when rats were approaching reward sites (9, 21.9 %), after arrival at reward sites (12, 29.3%) or spanning both phases (20, 48.8 %). A majority of responses signaled reward presence (25, 61.0%) whereas only three (7.3%) neurons responded when the wells were found unbaited. Four (9.8%) other neurons showed firing rate

changes in both conditions with different response magnitudes. Firing rate changes in the remaining group of neurons (9, 21.9%) reached significance only when the reward presence and absence condition where lumped together. Responses could be generated in relation to one (10, 24.4%), two (6, 14.6%) or all three reward sites (3, 7.3%). When a neuron responded to several reward sites, differences in magnitude were observed between the individual responses (5 out of 9 neurons, 55.6%). Changes in the firing rate of about half of the responding neurons (22, 53.7%) reached significance only when the three reward sites were pooled.

A large majority of responsive neurons fired differentially for reward presence or absence, for the various reward sites or for both conditions (i.e. reward location and availability; 34, 82.9%), which renders the possibility that their firing was purely spatially modulated highly unlikely [8,9]. For two neurons which increased their firing rates to a single reward site irrespective of the presence or absence of a reward this option cannot be excluded. Reactivation analysis yielded similar results when these two neurons were removed from the data set.

Control procedures for reactivation of subgroups To examine whether the observed strong reactivation for the 'Both Modulated' , 'Double Correlates' and 'HC→ VS' groups was due to cell- and time-specific firing correlations, EV and REV values were recalculated after the binned spike trains of the track-running episode had been randomized in three different ways; i.e. BIN, SHIFT and SWAP randomization procedures (see above "Randomized controls for verification of cross-structural reactivation" for further details). After randomization of the spike trains, whether that was done using the BIN, SHIFT or SWAP method, reactivation values (EV-REV) of all three groups tested were abolished and the (EV-REV) distributions after randomization were significantly different from the original distribution for all comparisons (Mann-Whitney's U-test (MWU), $p < 1 \cdot 10^{-4}$).

In principle, differences in numbers of cell pairs and/or correlation distributions may account for the differences in the observed reactivation between the subgroups according to the 'Theta modulation', 'Expression of correlates' and 'Firing order' partitions. To examine these possible confounds the bootstrapped reactivation distribution (EV-REV) of the subgroup with the

highest reactivation in each partition (referred to as “test group”; i.e. ‘Both Cells’, ‘Double Correlate’ and ‘HC → VS’) was tested against the (EV-REV) distributions obtained from 1000 samples of each of the other subgroups in that partition, matched for the total number of cell pairs and showing a comparable distribution of correlation strengths (MWU: $p > 0.05$). In the ‘Theta modulation’, ‘Expression of correlates’ and ‘Firing order’ partitions, the sampled subgroups all showed significantly less reactivation than the ‘Both Cells’, the ‘Double Correlate’ and the ‘HC → VS’ groups (MWU, $p < 1 \cdot 10^{-4}$).

Another possibility is that the differences in reactivation between the test group and the other sub-groups in the respective partition are due to differences in spike counts. This possibility was examined by comparing the (EV-REV) distribution of the test group with distributions of 1000 samples of the other subgroups in the partition under the condition that numbers of cell-pairs and spikes matched. To ensure similar spike counts in each group, we first assessed the spike count differences between the test group and the sample separately for the HC and VS units and for each of the behavioral episodes (i.e. pre-behavioral rest, track running and post-behavioral rest). If the spike count in a specific cell type - episode combination was lower for the sample than for the test group, the difference in spike counts was extracted from the test group. The abundant spikes were randomly removed from the spike trains but equally distributed across cells. Despite comparable spike counts, reactivation in the ‘Both Modulated’, the ‘Double Correlates’ and the ‘HC → VS’ groups remained significantly stronger than in any of the subgroups belonging to the respective partition (MWU, $p < 1 \cdot 10^{-4}$).

In summary, the strong reactivation of the test groups can be attributed to cell and time specific firing patterns. The difference in reactivation between the test group, i.e. ‘Both Cells’, ‘Double Correlate’ and ‘HC → VS’, and the other subgroups in the partitions cannot be explained by a difference in number of cell pairs, distribution of correlation strengths and observed number of spike counts.

Further analysis of maintained firing order from active behavior to post-behavioral rest As pointed out in the main text, the large majority of HC-VS pairs with double correlates, which maintained their firing order from track running to post-behavioral rest showed hippocampal firing preferentially before ventral striatal firing. Although this result indicates a predominance of HC→VS pairs in replay, it might be the case that this effect arises from a lack of clear cross-correlations in which the VS precedes the HC during track running. To examine this possible confound, we analyzed all cell pairs exhibiting significant peaks in their cross-correlograms of the track running period (n = 222; see Experimental Procedures for assessment of significant peaks). In most of these cell pairs the hippocampal cell fired preferentially before the ventral striatal cell during track running (n=153, 69%). Activity of the ventral striatal cell preceded that of the hippocampal cell in 54 pairs (24%) and in the remaining 15 pairs (7%) the peak was found at 0 offset. Thus, although during behavior more highly cross-correlated HC→VS pairs were found than VS→HC pairs, there was no shortage of pairs in which the VS neuron fired before the HC neuron did. As in the subgroup of cell pairs exhibiting double correlates, the time-offsets of the significant peaks during track running were positively correlated to those in post-behavioral rest ($R^2 = 0.09$, $p < 0.005$, n = 84) but not to those of pre-behavioral rest (n = 64), which confirms the experience dependence of replay in this larger population of cells. Similarly, most of the cell pairs that showed significant peaks during track running and in post-behavioral rest maintained their firing order (72/84; 86%).

Cell pairs exhibiting a HC→VS firing order during track running showed a recurring peak with the same offset sign during post-behavioral rest in 58 out of 153 cases (38%). This fraction was almost twice as high as for pairs that showed a VS→HC order (11/54, 20%; Fisher exact $p < 0.02$). Similar results were obtained when the analysis was performed more selectively, viz. when only cell pairs were taken into account having cross-correlograms with significant peaks and containing more than 500 counts (within [-2000,2000] ms, binsize 50 ms) during track running. In conclusion, during behavior we found more HC→VS pairs with well-articulated cross-correlations than VS→HC pairs, but even if this difference is taken into account, the probability that a cross-

correlation with the same order recurs in the HC→VS group remained significantly higher than for the VS→HC group.

Intra-area reactivation occurs also in a forward direction during rest and sleep

Besides cross-structurally, reactivation occurred within the HC (EV: 16.9 ± 3.6%; REV: 4.5 ± 1.2%; n =21) and within the VS (17.6 ± 4.1%, REV: 5.2 ± 1.3%; n =30; see Fig S3). We explored whether in addition to HC-VS pairs, reactivating cell pairs within the HC and VS are also preserving their firing order from the track running period to the post-behavioral sleep. Cross-correlograms were constructed for HC-HC and VS-VS pairs exhibiting a double correlate for each rest/active episode. The offset sign (+/-) of the peaks was compared for active behavior and rest episodes for pairs that showed significant peaks in the cross-correlograms for track running and the post-behavioral rest episode. A total of 232 HC-HC pairs were considered of which 81 (34.9%) showed significant peaks in the cross-correlograms of running and post-behavioral rest. In 75 of the pairs the offset sign was similar in both episodes (75/81 = 92.6%; Sign test: $p < 10^{-24}$). In addition, 43 VS-VS pairs were analyzed. Of these pairs, 19 (44.2%) showed a peak in the cross-correlograms of track running and post-behavioral rest and the offset sign was similar in 14 pairs (14/19 = 73.7%; Sign test: $p < 0.0001$). These results indicate that reactivation during sleep is occurring in a forward direction also within the hippocampus and ventral striatum. For the hippocampus this finding is consistent with previous literature [2,10] whereas this result has not been reported before for the VS.

Assessment of pre-behavioral rest activity patterns in relation to task anticipation

Given the prominent role of the VS in reward anticipation [8,11,12] it is interesting to examine whether the contextual priming that may occur when rats enter the experimental room elicits firing patterns that reflect elements of the upcoming task. If such anticipatory reactivation would occur during the pre-behavioral rest phase, it is expected to build up over sessions because the track was novel to the rats at the first recording session (i.e. in the pre-behavioral rest of session 1, the rat does not have track running experience). A measure that would indicate anticipatory

reactivation is the correlation coefficient of the Pearson's correlation matrices of pre-behavioral rest and track running (i.e. the matrix-based correlation value $r_{Track,R1}$, see the Materials and Methods section "Quantification of reactivation") which assesses, per session, the similarity between the cell pair-based spike train correlations from the pre-behavioral rest phase and those from the track running phase without taking the post-behavioral rest correlations into account. If the rat would acquire an expectation of the task that is expressed in the correlated firing of neurons during pre-behavioral rest and reflects upcoming track running, the matrix-based correlation coefficients $r_{Track,R1}$ would positively correlate with increasing experience on the track. Using a regression model, we did not observe a statistically significant relation between the $r_{Track,R1}$ correlation coefficients and the number of sessions the rat had experienced on the track before (R^2 = -0.1, p = 0.2, n = 21), nor a rise in the $r_{Track,R1}$ correlation coefficient after the first session, indicating that a prominent occurrence of anticipatory reactivation is unlikely.

Supporting Discussion:

Variants of memory consolidation theory. General accounts of memory consolidation theory predict that the HC initiates reactivation processes within this structure itself, but also in projection areas [18-21]. However, these accounts have not always explicitly pointed out how this initiating role would be neurally implemented, or how the leading function of the hippocampus would be expressed in correlated neural activity. For the neocortical-hippocampal system, Qin et al. [13] hypothesized that a long, continuous stretch of a neural representation of an experience will be stored in the wakeful state, and that a forward replay of the same experiential information would take place during sleep, with the hippocampal activity replaying information prior to corresponding temporal segments of neocortical activity. Applying this scheme to the hippocampal-ventral striatal system, the sequence of neural activity during wakeful experience would be represented as:

Hippocampus: ....-A – B – C - D …..

Ventral striatum: ....-A´- B´– C´- D´…..

where A, B, C and D code for 4 consecutive events such as visits to adjacent locations in space, and A´, B´, C´ and D´ represent ventral striatal codes for different states pertaining to these locations, e.g. expected or consumed rewards. During sleep, a time shift selectively advancing hippocampal activity would lead to:

Hippocampus: .....-A – B – C – D …..

Ventral striatum: ……..-A´- B´– C´- D´…..

Note that in this scheme, the number of HC→VS cell pairs does not necessarily exceed the number of VS→HC cell pairs, because no clear difference will arise if A´ in the VS is preceded by other VS activity that is part of the same continuous stretch of experience.

However, for the HC-VS system there are various arguments against sleep replay of continuous or at least fairly long sequences. First, sleep replay in the HC itself appears mainly restricted to episodes of ripple-sharp wave activity [14]. Second, the mean firing rate of VS neurons is enhanced for about 0.5 s following ripple onset, but depressed before onset [15]. Third, cells of which the firing rate is modulated by hippocampal ripples reactivate more strongly than non-modulated cells, and VS reactivation is stronger during a time window of about 200 ms following ripple onset than during time intervals in between ripples [12]. Altogether, these arguments corroborate the idea that replay in VS and HC is mainly restricted to (peri-) ripple episodes**.** Because it can be reasonably assumed that ripples originate in the hippocampal formation, and because VS cells are intrinsically hyperpolarized and need abundant convergent input to approach firing threshold [16], hippocampal firing will occur early after ripple onset [17,18] whereas some time will elapse before VS neurons become sufficiently excited to fire, which results in the following replay sequence during sleep:

Hippocampus: [A – B – C]

Ventral striatum: B´– C´- D´......

Note that the length of the hippocampal replay segments is delimited by boundaries of ripples or bouts of ripples (marked as [...]), whereas the element A´ is omitted from the VS replay sequence because of the delayed VS excitation relative to ripple onset. As already noted, VS excitability remains enhanced for a brief period after ripple offset, hence the maintenance of VS element D´ following ripple termination. Both phenomena will contribute to a higher proportion of HC→VS pairs than VS→HC pairs during sleep replay.

This variant of consolidation theory is well compatible with the temporal compression as observed (Fig.3). Moreover, this ´segmental´ form of replay does not need to result in loss of the A´ element originally coded by the VS, because A´ will be part of other segments in which it occupies a later position than in the example given above (e.g. X´ - Y´ - Z´ - A´). In conclusion, the result that more HC→VS than VS→HC pairs contribute to sleep replay is not only consistent with consolidation theory in general, but also confirms a specific, plausible variant.

References

1. Ji D, Wilson MA (2007) Coordinated memory replay in the visual cortex and hippocampus during sleep. Nat Neurosci 10: 100-107.

2. Lee AK, Wilson MA (2002) Memory of sequential experience in the hippocampus during slow wave sleep. Neuron 36: 1183-1194.

3. Diba K, Buzsaki G (2007) Forward and reverse hippocampal place-cell sequences during ripples. Nat Neurosci 10: 1241-1242.

4. Jackson JC, Johnson A, Redish AD (2006) Hippocampal sharp waves and reactivation during awake states depend on repeated sequential experience. J Neurosci 26: 12415-12426.

5. Quirk MC, Wilson MA (1999) Interaction between spike waveform classification and temporal sequence detection. J Neurosci Methods 94: 41-52.

6. Schmitzer-Torbert N, Jackson J, Henze D, Harris K, Redish AD (2005) Quantitative measures of cluster quality for use in extracellular recordings. Neuroscience 131: 1-11.

7. Louie K, Wilson MA (2001) Temporally structured replay of awake hippocampal ensemble activity during rapid eye movement sleep. Neuron 29: 145-156.

8. Roitman MF, Wheeler RA, Carelli RM (2005) Nucleus accumbens neurons are innately tuned for rewarding and aversive taste stimuli, encode their predictors, and are linked to motor output. Neuron 45: 587-597.

9. Lavoie AM, Mizumori SJ (1994) Spatial, movement- and reward-sensitive discharge by medial ventral striatum neurons of rats. Brain Res 638: 157-168.

10. Skaggs WE, McNaughton BL (1996) Replay of neuronal firing sequences in rat hippocampus during sleep following spatial experience. Science 271: 1870-1873.

11. Schultz W, Apicella P, Scarnati E, Ljungberg T (1992) Neuronal activity in monkey ventral striatum related to the expectation of reward. J Neurosci 12: 4595-4610.

12. Lansink CS, Goltstein PM, Lankelma JV, Joosten RN, McNaughton BL, et al. (2008) Preferential reactivation of motivationally relevant information in the ventral striatum. J Neurosci 28: 6372-6382.

13. Qin YL, McNaughton BL, Skaggs WE, Barnes CA (1997) Memory reprocessing in corticocortical and hippocampocortical neuronal ensembles. Philos Trans R Soc Lond B Biol Sci 352: 1525-1533.

14. Kudrimoti HS, Barnes CA, McNaughton BL (1999) Reactivation of hippocampal cell assemblies: effects of behavioral state, experience, and EEG dynamics. J Neurosci 19: 4090-4101.

15. Pennartz CM, Lee E, Verheul J, Lipa P, Barnes CA, et al. (2004) The ventral striatum in off-line processing: ensemble reactivation during sleep and modulation by hippocampal ripples. J Neurosci 24: 6446-6456.

16. Pennartz CM, Groenewegen HJ, Lopes da Silva FH (1994) The nucleus accumbens as a complex of functionally distinct neuronal ensembles: an integration of behavioural, electrophysiological and anatomical data. Prog Neurobiol 42: 719-761.

17. Buzsaki G (1986) Hippocampal sharp waves: their origin and significance. Brain Res 398: 242-252.

18. Ylinen A, Bragin A, Nadasdy Z, Jando G, Szabo I, et al. (1995) Sharp wave-associated high-frequency oscillation (200 Hz) in the intact hippocampus: network and intracellular mechanisms. J Neurosci 15: 30-46.
